# Fecal thyroid hormone metabolites in wild ungulates: a mini-review

**DOI:** 10.3389/fvets.2024.1407479

**Published:** 2024-05-22

**Authors:** Valeria Pasciu, Maria Nieddu, Francesca Daniela Sotgiu, Elena Baralla, Fiammetta Berlinguer

**Affiliations:** ^1^Department of Veterinary Medicine, University of Sassari, Sassari, Italy; ^2^Department of Medicine, Surgery and Pharmacy, University of Sassari, Sassari, Italy

**Keywords:** wild ungulates, fecal, thyroid hormone, environmental variables, individual variables, FTMs assay

## Abstract

This review aims to analyse the fluctuations of fecal thyroid hormone metabolites (FTMs) related to environmental and individual variables in different species of wild ungulates and provide a collection of assay methods. The great advantage of fecal sampling is being completely non-invasive. A systemic search was conducted from 2019 to 2024, using data sources PubMed, Scopus, Web of Science, and the World Wide Web, and ten studies were found on this topic. Three studies used the radioimmunoassay method for FTMs analysis, while the others used a less expensive enzyme-linked immunosorbent assay. Most of these papers validated the method for the species-specific matrix. Related to the studied variables, some authors analysed FTM fluctuations only concerning individual variables, and others in response to both. Temperature and fecal cortisol metabolites (FCMs) were the most studied environmental and individual variables, respectively. Since FTMs are an integrative measure of plasma thyroid hormones, the information obtained from a non-invasive-assay method regarding wild ungulate physiology is becoming of great interest to the scientific community.

## 1 Introduction

Thyroid gland function and activity of thyroid hormones (THs) are considered crucial to animal physiological functions. THs act on many different target tissues, stimulating oxygen utilization and heat production in every cell of the body. The overall effects are to increase the basal metabolic rate, to make more glucose available to cells, to stimulate protein synthesis, to increase lipid metabolism and to stimulate cardiac and neural functions (1). THs are also the primary endocrine regulators of body temperature (2). They can be considered indicators of the metabolic and nutritional status of the animals since allow them to adapt their metabolic balance to different environmental conditions, variations in nutrient requirements and availability, and to homeorhetic changes during different physiological stages.

Considering the growing need of monitoring wild animal populations, the determination of the seasonal fluctuations of THs can provide valuable insights into their response to environmental changes and ability to adapt to harsh conditions. Wild animals are more exposed to unpredictable changes in their living environment, and they can implement various physiological mechanisms in response to such changes (3, 4), such as increasing energy demands or reducing energy turnover (5).

For this reason, in the last few years, analytical methods to assess the concentration of hormones and metabolites in alternative biological matrices, such as feces (3, 6–9) and hairs (10, 11) have been intensively developed. A non-invasive approach is an animal-friendly technique that allows easy access to a great number of samples in a larger spatial–temporal window and provides a measure of hormone concentrations over a longer period (12). This is particularly useful in wild animals avoiding the need to capture and restrain them, thus reducing the stress suffered (13) and respecting animal welfare (6, 8, 14, 15).

The excretion of THs mainly occurs through the bile, and this offers the possibility of assaying them and their metabolites in fecal samples (6, 7, 16–18). Given that fecal thyroid hormone metabolites (FTMs) concentrations reflect the plasmatic levels of the biologically active T3 hormone (15), in recent years, several analytical methods for FTMs assaying in wild ungulates have been developed (3, 7, 19, 20). FTMs are a great indicator of the metabolic and energetic responses of wild animals to environmental factors (3, 7). Moreover, they are successfully used to obtain information on physiological animal status as pregnancy, lactation, age, and sex (6, 17).

Starting from these premises, this review aims to collect literature data on fluctuations of FTMs in wild ungulates in relation to individual or environmental variables, in order to identify common trends among species. The environmental variables are external factors linked to the animal’s habitat (e.g., external temperature and resource availability), while the individual ones are intrinsic characteristics of each animal, such as sex, age, weight, and body condition. Moreover, this review provided an overview of analytical methods for the FTMs assay. A complete literature search was carried out in PubMed, Scopus, Web of Science and the World Wide Web, using fecal/faecal thyroid hormones or fecal/faecal T3 in wild animals as keywords, and focusing on the ungulates species in the last five years (2019–2024). The ungulate species were classified according to previous studies (21–24).

## 2 Thyroid hormones (THs)

THs can be considered indicators of animal metabolic and nutritional status (1) by regulating basal metabolism, blood pressure, and body temperature (17, 25), and stimulating proteins, fat, and carbohydrate metabolism (17, 26). The THs circulating level is correlated with energy expenditure, body weight, and appetite (2).

**Figure 1:**
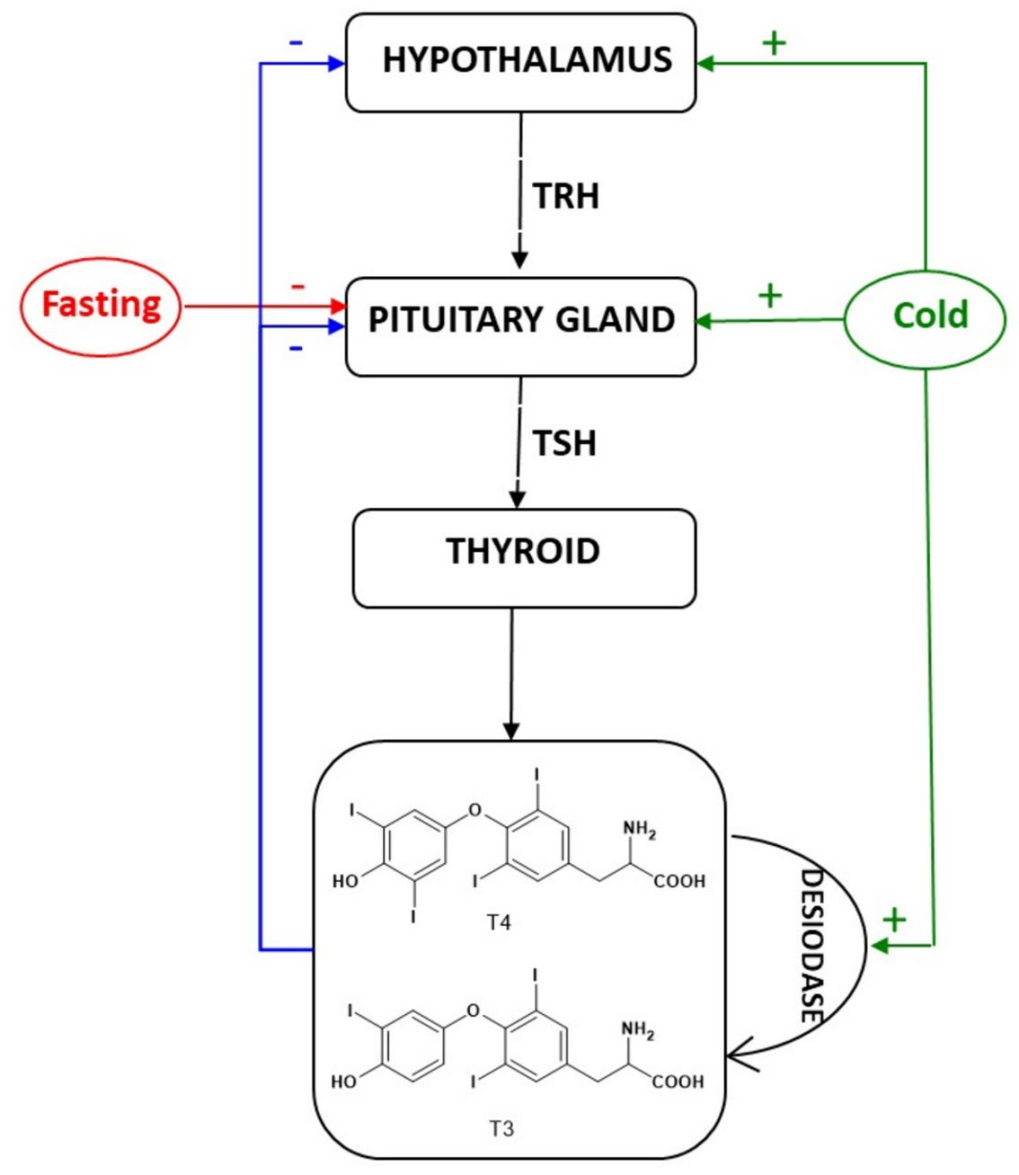
Hypothalamus-pituitary-thyroid system: production of thyroid hormones and the feedback effect.

As shown in Figure 1, THs are released by the thyroid gland under the regulation of the hypothalamus-pituitary-thyroid system (27). Thyrotropin-releasing hormone (TRH), produced and released by the hypothalamus, stimulates the anterior pituitary gland to secrete thyroid-stimulating hormone (TSH, or thyrotropin) in the bloodstream. In the thyroid gland, TSH stimulates the production of tetraiodothyronine (T4) and triiodothyronine (T3). Most of the circulating T3 originates at peripheral levels from T4 conversion; T3 is more biologically active and potent than T4 (28) but the latter has a longer blood half-life (29). It enters the cell through molecular transporters and binds thyroid receptors (TRs), which classically act as transcription factors (30).

Moreover, negative feedback is exerted by T4 and T3 that control the further secretion of TRH and TSH, through long and short feedback loops respectively, to maintain physiological levels of THs hormones (31).

THs also play an important role in adaptation to changes in environmental temperature (32). It was shown that exposure to cold temperatures increases serum T3, which has been identified as an outcome similar to that observed in hyperthyroidism (33, 34). The increased of T4 and T3 levels, after cold adaptation were confirmed by different authors (32, 35, 36). The cold temperature would act at the level of the hypothalamus or pituitary gland to increase TSH concentration and stimulate T3 and T4 production (32). The cold adaptation also causes deiodination of thyroxine (T4), acting on desiodase enzyme, and thus promotes an increase in blood T3 levels in humans and animals (32).

Moreover, as it is evident from the diagram in Figure 1, fasting also influences THs production. During the fasting period, TSH decreases with a consequent decrease of T3 and T4 (37). Furthermore, the prolonged fasting causes a decrease in muscle mass (2). Overall, the physiological response of animal at starvation is a decrease of THs production, operating on pituitary gland, to keep the basal metabolic rate. Therefore, as consequence of nutritional deficit, the metabolism tends to slow down, allowing the body to conserve its energy. Given the correlation between THs and body fat, the THs can be considered as an index of body condition (38), helping to discriminate between nutritional stress and other sources of stress (39).

## 3 FTMs fluctuation related to environmental or individual variables in wild ungulates

The excretion of THs through the bile created the favourable conditions for the determination of FTMs, that reflect the plasmatic levels of biologically active T3 (15), with the great advantage of a sample collection completely non-invasive. Additionally, since FTMs are an integrative measure of plasmatic THs, they represent an average value over the previous 24–48 h, as reported by Chizzola et al. for impala (*Aepyceros melampus*) (40).

A careful review of the literature over the past 5 years, showed ten manuscripts on FTMs fluctuations in wild ungulates about environmental and individual variables (Table 1). Seven studies analyzed FTMs levels in response to both variables, while the other three investigated this correlation only relating to individual variables.

**Table 1:** Monitoring of FTMs in wild ungulates.

Species	Geographic region	Analytical procedures				Investigated variables		FTMs concentration range (ng/g)	Year	Ref.
		Solvent extraction	Method	Commercial kit	Validation method	Environmental variables	Individual variables			
Vicuña (*Vicugna vicugna*) Free-ranging	Colombia (south America)	70% ethanol	RIA	(125I-total-triiodothyronine, MP Biomedicals, Orangeburg, NY)	Not reported	Behavioral response Anthropogenic disturbances	FCMs	16–246	2020	(41)
Impala (*Aepyceros melampus*) Free-ranging	Tanzania (Eastern and Southern Africa)	80% methanol	RIA	(125I-total-triiodothyronine MP Biomedicals, Costa Mesa, CA)	Analytical Biological	Temperature Food quality Anthropogenic disturbances	Sex Nutritional status FCMs	414–1,358	2020	(3)
African elephant (*Loxodonta africana*) Free-ranging	Pretoria state (South Africa)	80% ethanol	ELISA	EIA-Kit for fecal (Ann Arbor, United States)	Analytical Biological	NDVI (Normalized Difference Vegetation Index) Temperature	Age Sex	590–600	2020	(42)
Musk deer (*Moschus berezovskii*) Free-ranging	Sichuan (China)	90% ethanol	ELISA	Bovine kit (Reagent Genie Ltd., Ireland).	Not reported	Birth weaning	Intestinal microbiota	45–85	2021	(43)
Tibetan antelope (*Pantholops hodgsonii*) Free-ranging	Qinghai-Tibet (China)	70% ethanol	ELISA	Detect X® T3 Immunoassay-kit (Arbor Assays)	Analytical Biological	None	Intestinal microbiota	Not reported	2021	(44)
European mouflon (*Ovis aries musimon*) Captivity	Sardinia (Italy)	70% ethanol	ELISA	Human Kit (DiaMetra Srl, Boldon, UK).	Analytical Biological	Temperature	Sex	17.05–46.45	2022	(7)
Asian elephant (*Elephas maximus*) Captivity Free-ranging	Zoo facilities (United States) Wasgamuwa National Park (Sri Lanka)	100% methanol	ELISA	EIA-kit (Ann Arbor, MI)	Analytical Biological	None	Musth	35.64–35.01	2022	(45)
Wild boar (*Sus scrofa*) Free-ranging	Changbaishan Mountains (China)	70% ethanol (17)	RIA	MP Biomedicals (Orangeburg, NY) (17)	Not reported	None	Parasites Nutritional status FCMs	Not reported	2022	(46)
Asian elephant (*Elephas maximus*) Captivity	Zoo facilities (United States)	100% methanol	ELISA	EIA-kit (Ann Arbor, MI)	Analytical Biological	Social environment within zoos	Musth Age Body condition	35.64–35.01	2023	(47)
Iberian red deer (*Cervus elaphus hispanicus*) Free ranging	National reserve of boumort (France)	55% methanol	ELISA	EIA Kit (IBL International, Hamburg, Germany).	Analytical	Seasonality: Temperature Solar irradiance Precipitation Anthropogenic disturbances	FCMs Nutritional status	20–120	2024	(39)

FCMs, Fecal Cortisol Metabolites; FTMs, Fecal T3 Metabolites; ELISA, Enzyme-linked-Immunosorbent Assay; RIA, Radioimmunoassay.

The temperature was the most investigated among the environmental variables (*n* studies = 4), followed by anthropogenic disturbances (*n* = 3). Fecal cortisol metabolites (FCMs) (*n* = 4), sex (*n* = 3), nutritional status (*n* = 3), age (*n* = 2) intestinal microbiota (*n* = 2), and musth (*n* = 2) were the most studied among the individual variables.

For FTMs monitoring, only three authors used RIA methods, while the others preferred immune-enzymatic methods. For FTMs extraction, the most commonly used solvent was ethanol. Furthermore, almost all methods were analytically and biologically validated for the species-specific fecal matrix, as summarized in Table 1.

The FTMs concentration ranged from a minimum of 16 to a maximum of 1,358 ng/g feces. This great gap could be related not only to the different used analytical procedures but also to various species and to studied physiological and environmental conditions.

In general, in examined manuscripts, FTM levels were inversely related to temperature, with higher values in colder periods (3, 7, 39), as physiologically expected. Moreover, FTMs increased in younger subjects and decreased with age for all reviewed cases (42, 47). The relationships between FTMs and FCMs (3, 39, 41, 46), and the characteristics of the intestinal microbiota (43, 44) showed contradictory results. Concerning parasites (46), a negative correlation was found. No correlation between FTM levels with sex (3, 7, 42), food quality (3), animal behaviour (41), anthropogenic disturbance (3, 41) and body condition (47) was found.

## 4 Discussion

Despite the pivotal role played by THs in animal physiology and their ability to cope with changes in environmental conditions, the literature on FTMs fluctuations in wild ungulates is limited. It is worth noting that in the last five years, among wild ungulates, THs blood levels were only analysed in African elephants (*Loxodonta africana*) and Asian elephants (*Elephas maximus*) of North American zoos, to correlate their fluctuations with intestinal microbiome composition (48). In previous years, other authors assayed THs hormones in serum or plasma of wild ungulates [for example mule deer (*Odocoileus hemionus*) (38) and on red deer (*Cervus elaphus*) (49)], but the majority of the studies on THs rely indeed on the use of an alternative matrix, as feces, that not involve the capture of animals and without affecting their welfare. In the last five years, ten articles were found in literature about this topic in wild ungulates. The investigated species were limited to some phylogenetic branches, as order *Proboscidea* family *Elephantidae*, and order *Artiodactyls* suborder *Ruminants* family *Cervidae, Camelidae* and *Bovidae, and* suborder *Suina* family *Suidae*. In the previous quinquennial (2014–2018) only three articles were published, two on forest musk deer in 2016 (19) and 2018 (20) and one on Alces in 2017 (50). Although the number of studies on this topic has increased in the last five years, the results strongly highlight the need to broaden this area of research to include a larger number of species.

In the reported studies, FTM fluctuations were investigated both in relation to individual and environmental variables. Among the first, age, sex, and nutrition were investigated (3, 7, 39, 42, 46, 47). Regarding the effect of age on FTM concentrations, the results agreed in finding higher levels in juveniles compared to adults (42, 47). Age-related differences in THs concentrations are well described in domestic ungulates, with the highest values found in neonates and the lowest in elderly animals (1, 6). These data are explained by the THs action in controlling metabolism that, during the growing period, and especially immediately after birth, must be higher to promote the individual’s growth (6, 20).

On the other hand, no variation between sexes (3, 7, 42) was found in wild ungulates. In general, the relation between FTMs and sex is controversial, because some authors reported FTMs concentration higher in male than female gender, and others vice versa (6, 8, 9, 20). These contradictory outcomes regarding sex could be probably explained with other variables, including environmental or age variables, but not with a gender-specific significance (50).

Only one study correlated the FTMs to body condition and three to nutritional status. No correlation was found for body condition (47), while contradictory results were found for nutritional status: one manuscript reported no correlation (3), one a negative correlation (46) and another one a positive correlation (39). Severe conditions such as increased/decreased precipitation or extreme temperature could be responsible for this variability (39). Certainly, the topic is of great interest and deserves further studies, considering that T3 might be interpreted as a measure of energy balance across a multitude of taxonomic groups (38).

The correlation between FTMs and FCMs has been investigated in four different studies (3, 39, 41, 46) with contradictory results. Based on previous findings, cortisol and THs concentrations are expected to be negatively correlated (27, 51, 52). However, Pritchard et al. (41) found no relationship between FCMs and FTMs in the vicuña, while Hunninck et al. (3) (in impala), Gort-Esteve et al. (39) (in Iberian red deer) and Liu et al. (46) (in wild boar) reported that FTMs did not decrease when fecal cortisol levels increased, showing instead a positive correlation.

It is important to highlight that two (40, 42) of these four studies did not validate the method for FTMs in the analyzed animal species, and this might not reflect exactly the real FTMs concentration, as declared by the same authors (41).

The possibility to discriminate between nutritional stress and other types of stress by comparing the levels of FTMs and FCMs is very interesting. This correlation should be in-depth investigated in the future for wild species, not only for ungulates, giving attention to the validation of the assay method, synonymous of data reliability and reproducibility.

Regarding environmental variables, the most investigated was the environmental temperature (3, 7, 39, 42). The results agreed with finding an increase in FTMs at lower temperatures and a decrease at higher temperatures. In general, the results found in reviewed ungulates for temperature were similarly observed in other animal species (20), confirming a common trend in FTMs fluctuations (2, 11, 12). Only one exception was reported by Gort-Esteve (39) who found a positive correlation between FTMs and cold temperature, explained by the same authors with a high nutritional stress in the winter period. Another studied environmental variable was anthropogenic disturbance (3, 39, 41), for which there was no correlation with FTMs, except for the impala (3). Hunninck et al. reported that human disturbance affected FTM levels when temperature was accounted for Hunninck et al. (3). As shown in Table 1, other environmental variables were investigated in single studies, such as behavioral responses (vigilance and foraging) (41), social environment within zoos (47), Normalized Difference Vegetation Index (NDVI) (42), birth weaning (43), solar irradiance and precipitations (39). Further studies are needed to allow more in-depth comparisons.

Regarding assay methods, only three papers (3, 41, 46) used a RIA method, while the others (7, 39, 42–45, 47) were all enzyme immunoassays. This trend can be explained considering that, although RIA test offers many advantages in terms of specificity and sensitivity, it is more expensive, requires specialized staff and its use could be dangerous to human health.

Generally, the sample pretreatment involves a preliminary freeze-drying, followed by a liquid extraction with solvents such as methanol and ethanol, in variable percentages between 55 to 100% Several authors reported that decreasing the percentage of ethanol to 70% increased the extraction efficiency (7), as also reported for different avian and mammalian species (17).

Most of these papers performed an analytical validation of the method, species-specific for the investigated matrix. This is of great importance to confirm the reliability of the reported data. Three research groups among the reviewed ones did not perform an analytical validation; between them, one used a kit already validated for a phylogenetically related species (43), another one used a kit for mammalian (41), while the third used a kit previously validated for feces of different species (46).

However, it must be emphasized that data obtained by a non-specifically validated method, mainly if the data themselves are controversial with that reported in the literature, could not reflect the true concentrations of FTMs (41).

The main problem of this non-invasive assay method is the long sample preparation (lyophilization and extraction), but the benefits far outweigh its disadvantages. The preservation of animal welfare, by reducing the stress associated with sample collection, represents one of the principal advantages of FTMs monitoring. It permits obtaining important information on wild ungulates physiology and health, and the degree of adaptation to environmental conditions. This information is becoming significant and of great interest to conservationists and the scientific community.

In conclusion, the non-invasive FTMs assay can represent a promising tool to study the response of animals, not only wild species, to environmental changes and their adaptation capability, representing an interesting physiological indicator for the future.

## Author contributions

VP: Conceptualization, Data curation, Formal analysis, Investigation, Writing – original draft, Writing – review & editing. MN: Data curation, Formal analysis, Writing – review & editing. FS: Writing – review & editing. EB: Writing – review & editing. FB: Data curation, Formal analysis, Funding acquisition, Writing – review & editing.
